# The mediating role of self-efficacy between workplace violence and PTSD among nurses in Liaoning Province, China: A cross-sectional study

**DOI:** 10.3389/fpsyg.2023.1090451

**Published:** 2023-02-23

**Authors:** Jiachen Lu, Yingying Yu, Bin Wang, Yanni Zhang, Haoqiang Ji, Xu Chen, Meng Sun, Yuxin Daun, Yuanping Pan, Yunting Chen, Yaohui Yi, Xiaofeng Dou, Ling Zhou

**Affiliations:** ^1^School of Public Health, Dalian Medical University, Dalian, China; ^2^Department of Nursing, The First Affiliated Hospital of Dalian Medical University, Dalian, China; ^3^Laboratory Animal Center, Affiliated Zhongshan Hospital Dalian University, Dalian, China; ^4^School of Public Health, Shandong University, Jinan, China

**Keywords:** PTSD, self-efficacy, workplace violence, nurses, China

## Abstract

**Purpose:**

Nurses are at high risk for workplace violence, which can lead to psychological problems. The purpose of this study was to determine the relationship between workplace violence, self-efficacy, and PTSD, and to further explore whether self-efficacy mediates the relationship between workplace violence and PTSD among Chinese nurses.

**Materials and methods:**

This cross-sectional study was conducted in Liaoning Province, China in 2020. A total of 1,017 valid questionnaires were returned. Each questionnaire included the Workplace Violence Scale, the General Self-Efficacy Scale, the Post-traumatic Stress Disorder Scale (PTSS-10), and demographics information. A hierarchical multiple regression approach was used to explore the mediating role of self-efficacy in the relationship between workplace violence and PTSD. The mediation model was then tested by the PROCESS macro in SPSS.

**Results:**

A total of 1,017 nurses were included in this study, and the average score of PTSD among Chinese nurses was 26.85 ± 13.13 (mean ± SD). After further adjustment for control variables, workplace violence was positively associated with PTSD, explaining 13% of the variance. High self-efficacy was associated with low PTSD, explaining 18% of the variance. Self-efficacy partially mediated the role of workplace violence and PTSD.

**Conclusion:**

The high scores of PTSD among Chinese nurses demand widespread attention. Workplace violence is an important predictor of PTSD in nurses. Self-efficacy is a significant factor in improving PTSD in nurses and mediates the relationship between workplace violence and PTSD. Measures and strategies to improve self-efficacy may mitigate the effects of workplace violence on PTSD in nurses.

## 1. Introduction

The World Health Organization (WHO) defines workplace violence (WPV) as abuse, threats, or assaults against employees in work-related settings or on their way to and from work, which involve explicit or implicit challenges to their safety, well-being, or health (WHO, 2021). Most nurses in both developed and developing countries are subjected to different types of violence (Liu et al., 2019a), which can not only harm them physically and psychologically but also jeopardizes the effectiveness of the health system (Kim, 2020; Hilton et al., 2022). WPV has long been recognized as a global problem, and medical personnel, especially nurses, are among the professionals that are most vulnerable (Sun et al., 2021; Aljohani, 2022). WPV from patients and visitors is a major occupational hazard for medical personnel (Aljohani et al., 2021).

The problem of WPV has continued to increase in recent years, and there is growing concern about nurses’ exposure to it (Babiarczyk et al., 2020; Shi et al., 2020). Studies have shown that 55.7 to 95.5% of nurses are usually exposed to at least one type of WPV (Havaei et al., 2020). Nurse in Ethiopia (Fute et al., 2015), South Korea (Park et al., 2015), Jordan (Al-Omari, 2015), Germany (Schablon et al., 2012), and Iran (Khoshknab et al., 2015) experienced rates of physical violence ranging from 18.22 to 56% and verbal abuse from 63.8 to 89.58% in the past 12 months, as well as sexual harassment, which ranged from 4.7 to 19.7%. Recently, there has been a general increase in this phenomenon, including in China (Lu et al., 2020).

WPV can lead to a number of serious consequences; studies have shown that it can negatively affect the physical and mental health of victims (Wang L. et al., 2022), and it is also associated with low job satisfaction and high turnover rates among healthcare workers (Akbolat et al., 2021). WPV can be associated with stress and distress, which in turn expose the victim to WPV (Magnavita et al., 2020). Furthermore, WPV is associated with both job burnout (Liu et al., 2019b) and mental fatigue (Wolf et al., 2017). Exposure to one or more types of WPV puts nurses at increased risk for mental health problems, which can eventually evolve into post-traumatic stress disorder (PTSD) and burnout (Kobayashi et al., 2020; Akbolat et al., 2021; Wang J. et al., 2022). In addition, WPV can reduce productivity (Ghavidel et al., 2019), work engagement (Tian et al., 2020), affect job performance, and negatively impact the therapeutic relationship (Duan et al., 2019). Although violence in hospitals directly harms nurses and hospitals, the ultimate victims are patients (Zhang et al., 2015; Njaka et al., 2020). As WPV increases, the hospital environment becomes unstable, which can lead to less autonomy for nurses, poorer relationships with physicians, more patients waiting to be placed, and a greater likelihood of nurses not giving medications on time or committing medication errors that can affect patients. Furthermore, WPV affects the perception of the profession, and medical professionals may not support their children entering the medical field, which may lead to a shortage of nursing staff in China (Yang and Gao, 2015; Liu et al., 2018).

Self-efficacy is the belief that one has the ability to make plans and take actions to achieve goals (Bandura, 1977). People with low self-efficacy have little motivation to take action and avoid difficulties. Nurses’ self-efficacy is a key predictor of how they function in terms of choosing behavior, effort expenditure, thought patterns, and emotional responses (Yao et al., 2018). Studies have shown that nurses with low self-efficacy cannot cope with stressful situations under high workload, which may lead to problems such as job burnout, anxiety and mental fatigue (De Simone et al., 2018).

PTSD is defined as an anxiety disorder caused by exposure to traumatic events. These events often involve serious threats of death or injury, to which the individual responds by feeling intense fear and helplessness (Meeting, 1994). Most people experience a traumatic event at least once in their lifetime, and some will develop PTSD. Some studies have shown that witnesses of traumatic events can develop PTSD symptoms even without direct involvement in the traumatic event (Ratrout and Hamdan-Mansour, 2020). There are at least two types of trauma that healthcare workers face on a regular basis (Shi et al., 2017a). The first type of trauma is physical assault or threats from a patient or a patient’s family (Al-Maskari et al., 2020), which is a form of WPV and is increasingly common in hospitals (Shi et al., 2017b). The second type of trauma comes from indirect sources, such as treating and witnessing a patient’s traumatic experience, death, or serious injury (Ratrout and Hamdan-Mansour, 2020). Therefore, PTSD is an increasingly serious and concerning problem among medical personnel (Petrie et al., 2018; Giorgi et al., 2020).

Some studies have shown that WPV and PTSD are related. A prospective study by Pihl-Thingvad showed that PTSD among social educators was positively associated with the frequency and extent of WPV (Pihl-Thingvad et al., 2019). Similarly, a review also showed a positive association between nurses’ exposure to WPV and PTSD (Wang J. et al., 2022). While some studies have confirmed the relationship between WPV and PTSD, less research has been done on the relationship between WPV, PTSD and self-efficacy. If self-efficacy plays an important role in WPV and PTSD, this may have important implications for nurse clinical practice, as the impact of WPV on nurse PTSD may vary depending on the nurse’s self-efficacy. In an experimental study on maternity, self-efficacy was shown to be negatively associated with PTSD (İsbir et al., 2016), with mothers with low self-efficacy having more severe postpartum PTSD symptoms. Self-efficacy has also been shown to play a mediating role in the perception of organizational support and PTSD symptoms among frontline healthcare workers in the outbreak of COVID-19 (Zhou et al., 2021). Based on the above literature, we hypothesized that self-efficacy plays an important role in nurses PTSD, and if self-efficacy is a mediating product between WPV and PTSD, it would provide an important direction for intervention to mitigate the adverse effects of WPV on PTSD. In conclusion, this article aimed to test three hypotheses of nurses: (1) Workplace violence is positively associated with PTSD; (2) self-efficacy is negatively associated with PTSD; and (3) self-efficacy mediates the relationship between WPV and PTSD.

### 1.1. Ethical approval and consent to participate

Ethical approval was obtained from the Ethical Committee of Dalian Medical University (Approval NO.: 2022-017). Each participant signed an informed consent form. Participants were informed of the purpose of the study prior to participation and were assured that their information was confidential. We confirmed that all the methods we used were in accordance with relevant guidelines and regulations.

## 2. Methods

This study was conducted from September 2020 to March 2021, relying on a cross-sectional design with a multistage random sampling method, in which one tertiary hospital was randomly selected from each of the five regions (east, west, south, north, and central) in Liaoning Province. As male nurses account for less than 1% of Chinese nurses, our study focused solely on female nurses (Yu et al., 2019). 240 nurses were randomly selected from each hospital to conduct the self-administered questionnaire survey, for a total of 1,200 nurses. All respondents signed an informed consent form prior to filling out the questionnaire. With the help of hospital staff, 1,200 questionnaires were distributed, and the study population included all nurses working full time in these hospitals with at least 1 year of experience. Interns were excluded from the study. 183 study participants were excluded because they did not complete the questionnaire due to time constraints or had data missing from the questionnaire. Thus, a total of 1,017 nurses were included in this study, with a participation rate of 85%. The study contained seven independent variables: age, household registration, monthly expenditure, sector of work, years of experience, WPV, and self-efficacy, totaling 20 entries. According to the requirements of multiple linear regression, the sample size should generally be 5–10 times the number of independent variable entries, and considering an 80% effective response rate, the minimum sample size required for this study was about 250 cases. In this study, as part of a large study, to explore the relationship between WPV and nurses’ mental health and reduce sampling error, we expanded the sample size as much as possible. Finally, 1,017 valid questionnaires were collected, which satisfied the minimum sample size required for the study.

### 2.1. Instruments

#### 2.1.1. Workplace violence scale

The WVS was used to measure the frequency of workplace violence experienced by the study participants in the past 12 months. The scale consists of five dimensions: physical assault, emotional abuse, threat, verbal sexual assault, and sexual assault. Each item ranged from 0 to 3, reflecting the frequency of participants’ exposure to WPV (0 = zero times, 1 = 1 time, 2 = 2 or 3 times, and 3 = more than 3 times). The total score is the sum of the items and ranges from 0 to 15, with higher scores implying more frequent exposure to WPV (Peek-Asa et al., 1998). WVS has been shown to have good reliability and validity among Chinese medical professionals, with a Cronbach coefficient of 0.92 in one study (Wang et al., 2006). In this study, the Cronbach coefficient was 0.80.

#### 2.1.2. General self-efficacy scale

The GSES consists of 10 items on a 4-point Likert scale (1 = not at all true, 2 = hardly true, 3 = moderately true and 4 = exactly true). The total score is the sum of the items and ranges from 10 to 40, with higher scores indicating higher self-efficacy (Shang et al., 2007). The Chinese version of the GSES has been confirmed to have good reliability, with a Cronbach coefficient of 0.87 in one study (Shang et al., 2007). In this study, the Cronbach coefficient was 0.90.

#### 2.1.3. Post-traumatic stress syndrome 10-questions inventory

The PTSS-10 is a 10-item self-report scale described in the Diagnostic and Statistical Manual, Fourth Edition (DSM-IV), for assessing symptoms of PTSD. Symptoms are rated on a scale of 1 (never) to 7 (always). The total score ranges from 10 to 70, with a higher score indicating the more severe PTSD symptoms (Weisæth, 1989; Stoll et al., 1999). The PTSS-10 showed good reliability in previous studies, with a Cronbach coefficient of 0.92 (Weisæth, 1989). In this study, the Cronbach coefficient was 0.94.

### 2.2. Sociodemographic variables

A total of five demographic variables were included in this study: age, household registration, monthly expenditure, sector of work, and duration of work. Age was divided into two categories: “<32 years” or “≥32 years.” Household registration categorized as “Liaoning Province” or “other provinces.” Monthly expenses were categorized as “<8,000 RMB” or “≥8,000 RMB.” Work departments were categorized as “ward,” “outpatient,” or “emergency.” Years of experience was divided into two categories: “<10 years” or “≥10 years.” The cutoff for age and monthly expenses categories is based on the median.

### 2.3. Statistical analyses

We used IBM SPSS Statistics 21.0 (IBM, Asia Analytics Shanghai) for statistical analysis. Two-sided *p*-values of <0.05 were considered statistically significant. Independent sample t-tests and one-way ANOVAs were used to test for between-group differences in continuous variables. Correlations between age, WPV, self-efficacy, and PTSD were examined using Pearson correlation analysis. Hierarchical multiple regression was conducted to analyze the significant influences of PTSD and the mediating role of self-efficacy in the relationship between WPV and PTSD. In the first step, all demographic variables (age, household registration, monthly expenditure, sector of work, and years of experience) were added to the model as control variables. In the second step, the independent variable WPV was added to the model. In the third step, the mediating variable self-efficacy was added to the model. In this study, the Variance Inflation Factor (VIF) value was less than 10, and there was no problem of multicollinearity.

The role of self-efficacy in mediating WPV and PTSD (Model 4) was tested in PROCESS macro (version 3.0 by Andrew F. Hayes) for SPSS based on a bias-corrected nonparametric percentile Bootstrap method (Hayes, 2013). All demographic variables were used as control variables, with WPV as the independent variable, PTSD as the dependent variable, and self-efficacy as the mediating variable. Prior to model testing, scores for WPV, self-efficacy, and PTSD were standardized. Total effects (path c), direct effects (path c′), and indirect effects (path a*b) were examined. Bias-corrected accelerated 95% confidence intervals (BCa 95% CIs) were calculated for the indirect effects. If the CI for the indirect effect did not contain zero, a mediating effect was considered to be present.

## 3. Results

The mean age of the participants was 33.27 ± 7.27 (mean ± SD), with 47.6% of the nurses being over 32 years old. 76.5% of the participants were from Liaoning Province, and 56.3% had monthly expenditures of less than 8,000 RMB. The highest percentage of nurses worked in wards (72.8%), and more than half of them (54.2%) had worked for less than 10 years. Among the five variables, monthly expenditure and work sector were significantly associated with PTSD, and PTSD symptoms were more severe in those with monthly expenditures of ≥8,000 RMB than in those with <8,000 RMB (*p* < 0.05). Nurses working in emergency departments had the highest scores for PTSD (*p* < 0.05). Age, monthly expenses, work sector, and years of experience were all relevant in terms of WPV (Table 1).

**Table 1:** Relationship between demographic characteristics and PTSD, self-efficacy, and WPV.

Variable	*N* (%)	Mean ± SD		
		PTSD	Self-efficacy	WPV
Age				
<32	533 (52.4)	26.55 ± 12.93	27.66 ± 4.06	5.98 ± 2.18
≥32	484 (47.6)	27.18 ± 13.35	27.45 ± 4.22	6.45 ± 2.62
*p*-value		0.22	0.78	**0.00**
Province				
This province	778 (76.5)	27.03 ± 13.13	27.52 ± 4.04	6.25 ± 2.48
Other provinces	239 (23.5)	26.28 ± 13.13	27.67 ± 4.41	6.04 ± 2.18
*p*-value		0.78	0.31	0.88
Monthly expenditure				
<8,000 RMB	573 (56.3)	25.86 ± 12.69	27.54 ± 4.17	6.08 ± 2.35
≥8,000 RMB	444 (43.7)	28.12 ± 13.57	27.58 ± 4.09	6.37 ± 2.48
*p*-value		**0.00**	0.43	**0.02**
Sector				
Wards	740 (72.8)	27.22 ± 13.43	27.59 ± 4.06	5.89 ± 2.12
Outpatient	154 (15.1)	24.24 ± 12.12	27.38 ± 4.90	6.78 ± 2.97
Emergency	123 (12.1)	27.88 ± 12.16	27.62 ± 3.51	7.36 ± 2.77
*p*-value		**0.02**	0.83	**0.00**
Years of experience				
<10	551 (54.2)	26.65 ± 12.51	27.58 ± 3.96	6.00 ± 2.16
≥10	466 (45.8)	27.09 ± 13.83	27.53 ± 4.33	6.45 ± 6.21
*p*-value		0.29	0.58	**0.00**

The bold value *p* < 0.05.

### 3.1. Correlations among continuous variables

There was a positive correlation between WPV and PTSD symptoms (*r* = 0.339, *p* < 0.05), a negative correlation between WPV and self-efficacy (*r* = −0.148, *p* < 0.05), and a negative correlation between self-efficacy and PTSD (*r* = −0.277, *p* < 0.05; Table 2).

**Table 2:** Correlations among age, WPV, self-efficacy, and PTSD.

	Mean ± SD	1	2	3	4
Age	33.27 ± 7.27	1			
WPV	6.20 ± 2.41	0.133*	1		
Self-efficacy	27.56 ± 4.13	−0.013	−0.148*	1	
PTSD	26.85 ± 13.13	0.008	0.339*	−0.277*	1

* *p* < 0.05.

### 3.2. Results of hierarchical multiple regression

In the first step of the hierarchical multiple regression, age, household registration, monthly expenditure, work sector, and years of experience were added to the model as control variables. After excluding the effects of the above control variables in the second step, WPV and PTSD were positively correlated (β = 1.940, *p* < 0.00), with WPV having a significant effect on PTSD, explaining 13% of the variance. In the third step, self-efficacy and PTSD were negatively correlated (β = −0.730, *p* < 0.00), and adding the mediating variable self-efficacy to the model explained an additional 18% of the variance in PTSD. The standardized regression coefficient of WPV decreased from 1.940 in the second step to 1.745 in the third step and was still statistically significant, tentatively indicating that self-efficacy partially mediated the relationship between nurse WPV and PTSD (Table 3).

**Table 3:** Hierarchical multiple regression analysis results for PTSD.

	Step 1	Step 2	Step 3
Step 1			
Age	0.868	0.446	0.228
Province	−0.426	−0.342	−0.258
Monthly expenditure	2.244**	1.601*	1.701*
Sectors	−0.161	−1.663**	−1.522**
Years of experience	−0.779	−1.122	−0.913
Step 2			
WPV		1.940**	1.745**
Step 3			
Self-efficacy			−0.730**
F	24.40**	17.89**	38.66**
Adjusted R^2^	0.00	0.12	0.17
△R^2^	0.01	0.13	0.18

* *p* < 0.05;

** *p* < 0.01.

**Figure 1:**
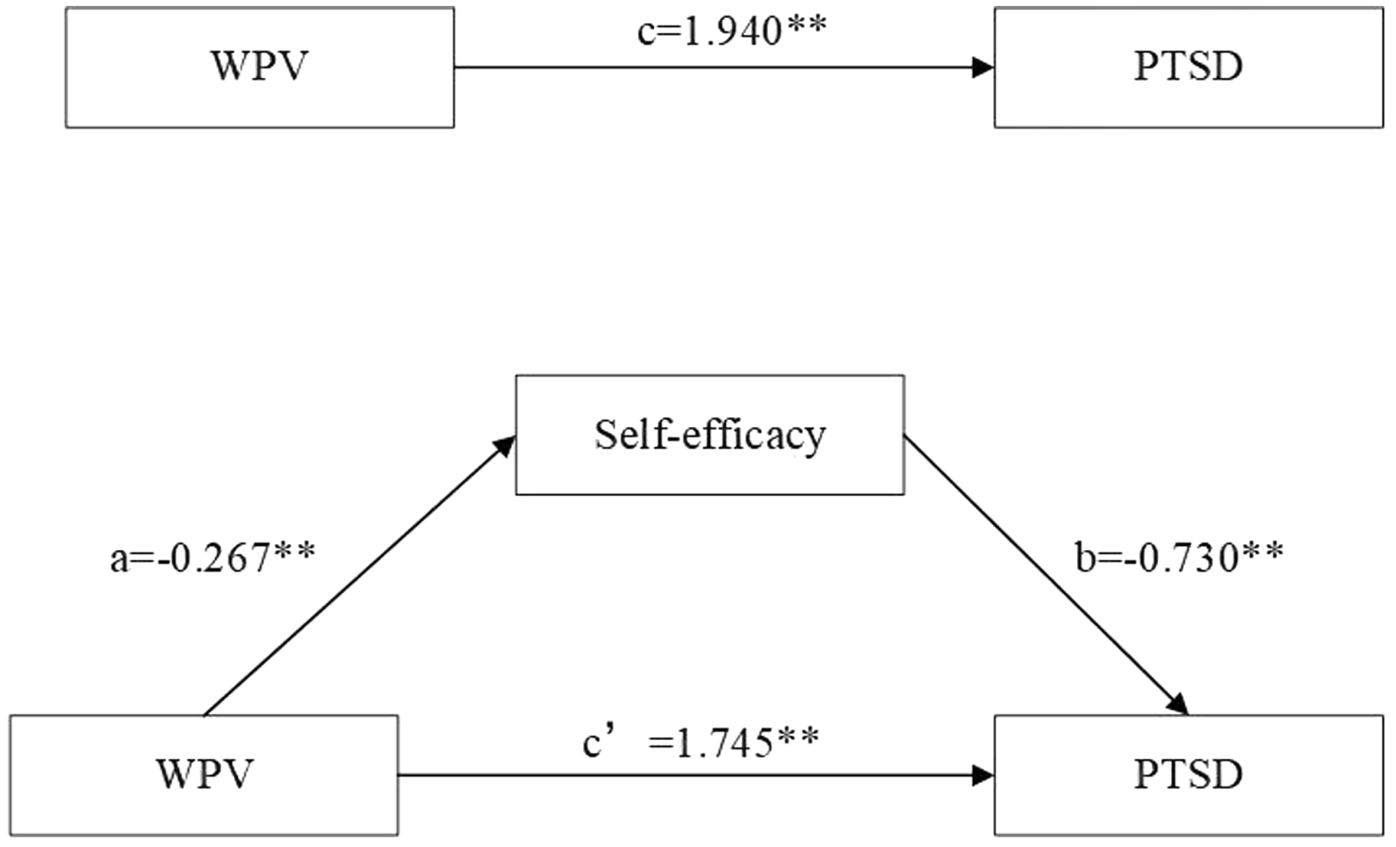
Model of the mediating role of self-efficacy between WPV and PTSD. **p < 0.01.

First, the relationship between WPV and PTSD was examined (path c). WPV and PTSD were positively correlated (c = 1.940, *p* < 0.00), and WPV had a significant indirect effect pathway on PTSD through self-efficacy (a*b, a = −0.267, b = −0.730, a*b (BCa 95% CI) = 0.195 [0.114, 0.293]). The confidence interval for the indirect effect did not contain 0, suggesting a mediating role of self-efficacy between WPV and PTSD. In addition, when self-efficacy was added to the model as a mediating variable, the direct effect of WPV on PTSD remained significant (c′ = 1.745, *p* < 0.00; Table 4). Therefore, self-efficacy played a partially mediating role in the relationship between WPV and PTSD. A visualization of the model is shown in Figure 1.

**Table 4:** Mediation analysis results.

Path	Coefficient/Effect	value of *p*	BCa 95% CI	
c	1.940	<0.01	1.616	2.263
a	−0.267	<0.01	−0.375	−0.159
b	−0.730	<0.01	−0.910	−0.550
a*b	0.195	--	0.114	0.293
c′	1.745	<0.01	1.427	2.062

BCa 95% CI: the bias-corrected and accelerated 95% confidence interval; age, household registration, monthly expenditure, sector of work, and years of experience were covariates; a: the direct effect of WPV on self-efficacy; b: the direct effect of self-efficacy on PTSD; a*b: the indirect effect of WPV on PTSD via self-efficacy; c′: the direct effect of WPV on PTSD; c: total effect of WPV on PTSD.

## 4. Discussion

Our study explored the relationship between WPV, self-efficacy, and PTSD and examined the mediating role of self-efficacy in the relationship between nurses suffering from WPV and PTSD. We found a positive correlation between WPV and PTSD and a negative correlation between self-efficacy and PTSD, with self-efficacy playing a mediating role in the relationship between WPV and PTSD. The average score of PTSD in this study was 26.85 ± 13.13 (mean ± SD), which was higher than that of other studies by some patients (sample size = 132; Milton et al., 2017), indicating a high level of PTSD among Chinese nurses, which should be of broad concern. Several studies have shown gender differences in the extent of PTSD, and female nurses tend to report higher levels of PTSD symptoms compared to male nurses, making the study of PTSD in female nurses even more important (Qi et al., 2022).

First, there was a positive correlation between WPV and PTSD, which is consistent with other studies (Zafar et al., 2016). Studies of WPV in various occupational groups have consistently indicated that WPV is associated with mental health (Rudkjoebing et al., 2020). Previous studies have shown that nurses are at very high risk of exposure to violence in the workplace, with three times the risk of experiencing WPV than any other occupational group (Kennedy and Julie, 2013). WPV affects the normal operation and reputation of hospitals, threatens the safety of medical staff, and acts as a stressor causing emotional distress to medical staff, which can develop into PTSD (Wang et al., 2015; Hilton et al., 2017). In a survey of more than 750 psychiatric hospital staff, 16% of participants met the screening threshold for PTSD on a self-report scale as a result of experiencing WPV (Hilton et al., 2020). Nurses with PTSD may experience physical distress such as headaches, insomnia, and anxiety, eventually leading to depression, absolute pessimism, or other psychiatric disorders (Mealer et al., 2009; Germain, 2013). In addition, nurses with PTSD are more likely to experience decreased productivity, burnout, medication errors, and lower overall quality of care (Lauvrud et al., 2009; Gates et al., 2011; Czaja et al., 2012). On the other hand, nurses with PTSD are at higher risk of WPV due to the fact that individuals with PTSD may be overly irritable, which can lead to interpersonal conflict and self-aggression (Kuijpers et al., 2012). Moreover, they may exhibit emotional numbness, making them less alert to environmental factors around them that signal danger, which may put them at increased risk of being targeted by their abusers (Chu, 1992), creating a vicious cycle. Research aimed at exploring ways to mitigate the effects of WPV on PTSD is critical, and we found that self-efficacy may fill this role.

Resilience can be considered a process, a trait, or outcome, but it can be broadly conceptualized as adaptation in the face of adversity (Southwick et al., 2014). Self-efficacy is widely associated with resilience, a form of cognition that promotes positive coping and adaptive responses to obstacles, thereby increasing resilience to traumatic environments (Connor and Davidson, 2003; Gallagher et al., 2020). In this study, we found a negative correlation between nurses’ self-efficacy and PTSD, which is consistent with other studies (Bosmans and van der Velden, 2017). In fact, high self-efficacy functions as a resilience factor among victims of different traumatic events. People with higher self-efficacy are less likely to experience PTSD (Cieslak et al., 2008; Guerra et al., 2018) because self-efficacy affects a person’s alertness to potential threats, and those who believe they can control those threats are less likely to feel distressed. In contrast, people with low self-efficacy are more likely to overestimate threats and worry about negative outcomes (Zhou et al., 2021). In previous studies, the protective effect of self-efficacy on PTSD symptoms was demonstrated for various traumatic experiences (Ginzburg et al., 2003; Gil, 2005).

More importantly, in this study, we found that self-efficacy can act as a mediator between WPV and PTSD symptoms, similar to the finding that self-efficacy can mediate between sexually abused adolescents and PTSD symptoms (Guerra et al., 2018). Previous studies focused more on the effect of self-efficacy on PTSD, whereas our study found that higher WPV may lead to lower self-efficacy and further lead to higher levels of PTSD symptoms. Self-efficacy is one of the cognitive factors that can explain the emotional response to traumatic situations. In this context, this factor refers to the individual’s belief in their ability to cope with the excessive demands caused by a traumatic event (Benight and Bandura, 2004). Although this factor has received little attention in the literature in relation to female nurse victims, theory suggests that self-efficacy can play a mediating role between traumatic events and the symptoms they cause. Specifically, it has been suggested that violent scenarios may reduce self-efficacy because the victim personally believes that they do not have the necessary resources to overcome the dilemma (Bandura, 1977; Benight et al., 1999; Diehl and Prout, 2002). Higher WPV leads to lower self-efficacy, which in turn is associated with high PTSD symptoms. These studies suggest that we can reduce PTSD symptoms in female nurses by reducing WPV and increasing self-efficacy; however, it is noteworthy that WPV still had a significant direct effect on PTSD, suggesting that self-efficacy is only a partial mediator, and there may be other variables that were not considered in this study.

Based on the results of this study, we propose the following recommendations in the hope of improving the severity of symptoms of PTSD in nurses and reducing the occurrence of WPV. First, the number of security guards should be increased and appropriate security measures should be improved in hospitals so that the risk of WPV can be reduced and victims can seek immediate professional help in the event of its occurrence. Second, we found that self-efficacy plays an important role in the relationship between WPV and PTSD and recommend that hospital administrators conduct health education activities about it and provide the necessary support for nurses. For the government, the health administration should improve the standardized management and guarantee system for nurse registration and promotion, as well as establish independent professional systems and academic organizations to increase nurses’ sense of belonging. For hospitals, nursing managers should adopt effective strategies to support nurses and give positive expectations to relieve their stress. Social persuasion is an effective measure to increase self-efficacy. Therefore, nurses who lack experience or support should be coached by confident, authoritative, and optimistic nurses to improve their confidence. In addition, simulation training in the department can effectively improve nurses’ self-efficacy, promote learning and communication among the team, and contribute to the improvement of nurses’ professional theories, skills, and problem-solving abilities. Finally, hospital support and care have the potential to reduce the harm caused by WPV to nurses, who should also be aware of all resources available in community homes for problem solving and maintaining good mental health (Shi et al., 2017b).

There are many studies on WPV in China, but most of them focus on its prevalence and influencing factors. As a stressor, WPV is closely relevant to PTSD, but there are not many related studies in China, especially for occupational groups such as nurses. This study is the first to explore the relationship between WPV and self-efficacy and PTSD among Chinese nurses, and to analyze the mediating role of self-efficacy. In this study, we found that WPV was an important predictor of PTSD for Chinese nurses; WPV was positively correlated to nurse PTSD, whereas self-efficacy was negatively correlated to PTSD. Self-efficacy mediated the relationship between WPV and PTSD. Measures and strategies to reduce WPV and improve nurse self-efficacy may reduce the occurrence of nurse PTSD. However, this study has some limitations. First, our data on workplace violence was collected retrospectively, an approach that relies on participants’ ability to recall events that occurred in the past 12 months, which can lead to recall bias. Second, the cross-sectional study could not draw a causal relationship between WPV, self-efficacy, and PTSD. Finally, this study was only conducted in Liaoning Province, and future research should expand the study population and region.

## 5. Conclusion

In conclusion, for nurses, WPV and PTSD were positively correlated, whereas self-efficacy and PTSD were negatively correlated. Self-efficacy mediated the relationship between WPV and PTSD. Strategies and measures to improve self-efficacy are expected to mitigate the effects of WPV on nurse PTSD.

## Data availability statement

The raw data supporting the conclusions of this article will be made available by the authors, without undue reservation.

## Ethics statement

Ethical approval was obtained from the Ethical Committee of Dalian Medical University. Each participant signed an informed consent form. Participants are informed of the purpose of the study prior to participation and are assured that their information is confidential. We confirmed that all the methods we used were in accordance with relevant guidelines and regulations.

## Author contributions

All authors made significant contributions to the conception and design, acquisition of data, or analysis and interpretation of data, participated in drafting the article or critically revising important intellectual content, agreed to submit the article to the current journal, gave final approval of the version to be published, and agreed to take responsibility for all aspects of the work.

## Conflict of interest

The authors declare that the research was conducted in the absence of any commercial or financial relationships that could be construed as a potential conflict of interest.

## Publisher’s note

All claims expressed in this article are solely those of the authors and do not necessarily represent those of their affiliated organizations, or those of the publisher, the editors and the reviewers. Any product that may be evaluated in this article, or claim that may be made by its manufacturer, is not guaranteed or endorsed by the publisher.

## Abbreviations

WPV: Workplace violence
PTSD: Post-traumatic stress disorder
WVS: Workplace Violence Scale
GSES: General self-efficacy scale
VIF: Variance inflation factor
SD: Standard deviation
